# Relationships Between Transformational Leadership and the Nursing Practice Environment: A Cross‐Sectional Study

**DOI:** 10.1155/jonm/9298780

**Published:** 2026-11-08

**Authors:** Marko Kaira, Anu Nurmeksela, Tarja Kvist

**Affiliations:** ^1^ Department of Nursing Science University of Eastern Finland Kuopio Finland uef.fi; ^2^ Nursing Research Center Cancer Center Helsinki University Hospital Helsinki Finland hus.fi; ^3^ Wellbeing Services County of Central Finland Jyväskylä Finland

**Keywords:** cross-sectional study, nursing practice environment, nursing quality, transformational leadership

## Abstract

**Introduction:**

Healthcare services require an adequate number of skilled healthcare personnel, an optimal practice environment, and the best possible leadership skills to ensure that the necessary services are provided in a high‐quality and economical manner. Hence, the aim of this study was to describe nursing staff perceptions of the nursing practice environment and transformational leadership, as well as explore whether they are related to each other.

**Methods:**

A quantitative cross‐sectional study involving 292 nurses from two Finnish university hospitals was conducted. The nursing practice environment was assessed using the Practice Environment Scale of Nursing Work Index, while transformational leadership was assessed using the Transformational Leadership Scale.

**Findings:**

The participating nurses rated the practice environment at 2.58 (out of 5), which reflects good conditions. None of the subscales associated with transformational leadership reached an excellent level, i.e., a score of ≥ 4 out of 5. All the PES‐NWI and TLS subscales showed statistically significant linear relationships. Nurses’ perceptions of participation in healthcare organizations were most strongly associated by the actions of nurse directors. The practice environment and transformational leadership were also found to be associated with nurses’ evaluations of overall quality of the nursing.

**Conclusion:**

The results suggested that nursing leadership in specialized healthcare must focus more on improving feedback and rewards, as these aspects received the lowest evaluations from the nurses. Also, nurse directors in Magnet‐aspiring hospitals should increase their interaction and visibility among nurses, as well as expand their role in organizations, if they wish to contribute to improving the practice environment. Strong transformational leadership and an excellent practice environment can promote the nursing quality.

**Implications for Nursing Management:**

The findings may help healthcare organizations and nurse leaders identify aspects of leadership and working conditions that warrant attention when developing the nursing practice environment and leadership practices.

## 1. Introduction

Comprehensive healthcare services are the foundation of a welfare society and necessary for ensuring good quality of life among citizens [1]. However, these services are currently facing significant challenges, including diminishing appeal of the profession, dependence on skilled personnel, and increasing financial pressure on publicly funded healthcare [2, 3]. Public healthcare systems must employ a sufficient number of competent healthcare professionals to ensure that citizens receive the services they need in a timely and high‐quality manner that is also economically sustainable. Enhancing leadership is of particular importance in modern healthcare, as the current practices tend to emphasize the administrative management of patient care while neglecting organizational culture, working conditions, and relational leadership [4]. Multiple meta‐analyses of nurse leadership have demonstrated a link between nurse leadership practices and outcomes such as work satisfaction, job commitment, care quality, and a healthy work environment [5, 6].

A potential solution to addressing the challenges affecting healthcare is the implementation of the Magnet Hospital Model, which was developed in the United States in 1980. The key components of this model include transformational leadership, empowering structures for staff, high‐quality professional practices, the utilization of new evidence‐based knowledge, and innovation; the combination of all these factors contributes to improved operational outcomes [7]. Magnet hospitals are characterized by greater nursing staff attraction and retention relative to non‐Magnet hospitals, and the implementation of the model is positively associated with both nurse and patient satisfaction [8, 9]. Finland has joined the Magnet hospital program, with two hospitals awarded this status in 2024 [10].

Although Finnish studies have examined the nursing practice environment and its associations with nursing outcomes [11–15], this topic has been more extensively studied on an international level [16–18]. Transformational leadership, on the other hand, has been widely studied both in Finland and on an international level [6, 19–26]. To the best of our knowledge, no prior research has investigated whether, and to what extent, transformational leadership influences the nursing practice environment, although there is research on the relationship between authentic leadership and the nursing practice environment [18, 27, 28].

In this study, the nursing practice environment refers to the conceptual framework defined by Professor Eileen T. Lake, which has been compiled into the Practice Environment Scale of the Nursing Work Index (PES‐NWI) instrument [29]. It is relevant to note that Lake [30] based her theoretical model of the nursing practice environment on sociology; as such, the theory explores how the complex operations of healthcare organizations can best be organized for the provision of high‐quality care. In hospitals, leaders are often required to make difficult decisions in the face of complex and unpredictable challenges while respecting employee autonomy. The nursing practice environment model includes the perspectives of both nurses and nurse leaders to demonstrate the key elements that promote the delivery of high‐quality care and enhance the attractiveness of healthcare organizations [29]. Nurse leaders who want to positively impact the organization in which they work can thus refer to this model to better understand which factors and structures are most relevant for their work.

Healthcare organizations, leaders, and employees can all use this practice environment framework to influence nursing operations and the practice environment to improve the work environment and the quality of care. This may reduce the likelihood that potentially poor decisions made by leaders will result in unfavorable outcomes in nursing practice [31]. Previous research has found that the quality of practice environments in the healthcare setting is associated with reduced workload among nurses, psychological stress, and intentions to leave a position; simultaneously, the quality of practice environments can increase job satisfaction and sense of accomplishment [32, 33]. Moreover, studies have also demonstrated how the practice environment can improve interprofessional collaboration and nursing care quality, as well as reduce missed nursing care, burnout, and work‐related stress [27, 33–35].

James MacGregor Burns [36] and Bernard Bass [37] first introduced the theory of transformational leadership, which includes four concepts related to the qualities of a transformational leader: idealized influence; inspirational motivation; intellectual stimulation; and individualized consideration [36, 37]. In the context of nursing, a transformational leader inspires employees to achieve better care outcomes and enhance their self‐leadership skills [38].

Nurse leaders who exercise transformational leadership have been found to provide numerous positive outcomes for nurses, patients, and healthcare organizations [25]. From the nurse’s perspective, transformational leadership from a nurse manager is associated with greater nurses’ resilience, structural empowerment, job satisfaction, commitment to work, and teamwork [28, 39, 40]. This leadership style is also positively associated with nurses’ psychological well‐being, as it has been shown to reduce burnout, psychological distress, and cynicism, while simultaneously increasing a sense of efficacy and psychological safety [6, 25, 28].

Transformational leadership among nurse leaders, when considered from a patient perspective, has shown a negative association with the occurrence of adverse events and a positive association with the quality of nursing care and medication safety [22, 41]. For instance, nurses who gave positive ratings of transformational leadership among nurse leaders also gave positive ratings of the provision of patient‐centered care [39, 40]. From the perspective of healthcare organizations, transformational leadership improves the safety culture within work units and the utilization of various quality systems. This leadership style has also been shown to reduce nurse turnover and increase employee commitment [28, 39, 40, 42].

The purpose of this study is to describe nursing staff perceptions of the nursing practice environment and transformational leadership, as well as explore whether they are related to each other. The goal of the conducted research is to produce research‐based knowledge that can be used to enhance the attractiveness of hospitals and improve the leadership skills of nurse leaders.

The study was guided by the following research questions:

• How do nursing staff perceive the nursing practice environment?
• Do the nursing staff perceive that nurse leaders exercise transformational leadership?
• What relationships exist between the nursing practice environment and the transformational leadership of nurse leaders?

## 2. Materials and Methods

### 2.1. Design, Samples, and Settings

The presented research followed a quantitative, descriptive cross‐sectional study design. A purposive total sampling approach was used and applied to specialized units at two Finnish Magnet‐aspiring university hospitals that employed a total of 4325 nurses. At the time of the data collection, there were no Magnet‐recognized hospitals in Finland; one hospital was on Magnet journey, and another hospital developed its work by the Magnet principles. As the target population was small and clearly defined, all eligible nurses in these units were invited to participate to ensure comprehensive coverage of the study population. In the absence of Magnet‐recognized hospitals in Finland, these units represent the closest approximation to the Magnet framework in the Finnish context, enabling the examination of the study phenomena within a theoretically relevant and emerging setting. Data were collected between February and March 2023, using an electronic Webropol survey questionnaire. A survey link, which included information about the study, consent for participation, and the questionnaire itself, was sent to the nurse director of participating units via designated organizational contact persons. The nurse director then forwarded the survey link to their staff, with one reminder sent out to potential survey respondents. A total of 292 nurses responded to the survey, resulting in a response rate of 7%.

### 2.2. Instrument

The questionnaire used in the presented research included eight background questions related to the respondent’s gender, organization, specialty, age, work experience, type of shift work, nursing role, and overall evaluation of nursing quality using a five‐point Likert scale (1 = poorest, 5 = best).

The quality of the nursing practice environment was assessed using the PES‐NWI [29], which measures five dimensions of the nursing work environment:

• Nurse participation in hospital affairs (9 items)
• Nursing foundations for the quality of care (10 items)
• Nurse manager’s ability, leadership, and support of nurses (5 items)
• Staffing and resource adequacy (4 items)
• Collegial nurse–physician relations (4 items)

Respondents scored the questions using a four‐point Likert scale: 1 = strongly disagree; 2 = disagree; 3 = agree; and 4 = strongly agree. The reliability of this instrument, including the subscales, has been demonstrated in several studies, with Cronbach’s alpha values ranging from 0.71 to 0.98 across the different subscales [14, 16, 18, 29]. A nursing practice environment was considered favorable when the mean score exceeded 2.5 [29].

Transformational leadership was assessed using the Transformational Leadership Scale (TLS), which was developed at the University of Eastern Finland [43]. The scale evaluates four aspects of a nurse manager’s actions:

• Ethical leadership (14 items)
• Management of the nursing process (16 items)
• Providing feedback and rewards (6 items)
• Support for professional development (7 items)

Additionally, the scale includes 11 items related to the actions of a nurse director. Respondents answered the questions using a five‐point Likert scale: 1 = strongly disagree; 2 = partially disagree; 3 = I cannot say; 4 = partially agree; and 5 = strongly agree. The reliability of the instrument, including the subscales, has been established based on the results of multiple Finnish nursing science studies, with Cronbach’s alpha values ranging from 0.878 to 0.968 across the different subscales [19, 21–24, 43]. Transformational leadership was considered excellent when the mean score was 4.0 or higher [19].

### 2.3. Ethical Considerations

A study information sheet, cover letter, GDPR‐compliant registry description, and impact assessment form were prepared, and research permissions were obtained from the participating organizations according to the specific approval procedures [44]. Participants were informed about the study, including that their participation is entirely voluntary, anonymity would be ensured, and that they have the option to withdraw at any stage of the study. Each respondent provided informed consent before answering the survey. Ethical review board statement was not required for this study according to Finnish ethical regulations [47].

### 2.4. Data Analysis

The collected data were reviewed and entered into IBM SPSS Statistics 27 (IBM, Armonk, NY). Missing data were not imputed. Cases with missing values for variables required in a particular analysis were excluded using listwise deletion. Consequently, the number of participants varied slightly across variables and analyses. Background variables were described using frequencies, percentages, mean and median values, and standard deviations. The reliability of the obtained mean values was tested using Cronbach’s alpha, while the distribution of mean scores was tested using the Kolmogorov–Smirnov test. The results were presented as mean values and standard deviations. The correlation between PES‐NWI and TLS subscales was tested using Pearson’s correlation coefficient, with a correlation coefficient of ≥ 0.1 indicating weak, ≥ 0.3 indicating a moderate, and ≥ 0.5 indicating strong relationship. The distributional assumptions of parametric analyses were assessed by examining skewness and kurtosis. The mean scores of subscales demonstrated acceptable approximation to normality. Also, Spearman’s rank correlations were computed as a robustness check. The results were consistent with Pearson correlations in terms of magnitude and statistical significance, and comparable coefficients can be seen in Results section [46].

The relationships between the scores for TLS subscales and each PES‐NWI subscale were further analyzed using multiple linear regression. In this analysis, the TLS subscales were used as explanatory variables and then tested individually against each PES‐NWI subscale. As a sensitivity analysis, linear mixed models were conducted to account for potential clustering at the hospital level. Exploratory comparisons of mean scores for both instruments across background variable groups were conducted using a one‐way ANOVA or the Kruskal–Wallis test combined with either an independent samples *t*‐test or Mann–Whitney *U*‐test; the decision as to which methods to use depended on variable distribution. A *p* value < 0.05 was considered to demonstrate a statistically significant difference [46, 47]. The study was reported in accordance with the STROBE guidelines.

## 3. Results

### 3.1. Demographics

When looking at the demographics of the respondents (*n* = 292), 87.3% were women and 9.2% were men. The average age of the respondents was 43.24 years, and the average work experience was 16.85 years. Furthermore, 66.1% of the respondents reported working shifts, 33.2% worked regular daytime hours, and 69.9% were registered nurses. Respondents’ gender and professional distribution were comparable to available nursing workforce statistics in Finland, that is, 92% women and 8% men; 50% are aged 36–55 [48]. However, detailed information on nonrespondents was not available. The respondents rated the overall quality of their unit’s performance as very good (*M* = 4.03) (Table 1).

**TABLE 1:** Demographic information of the nurses who participated in the study.

Variables (*n* = 292)	*n*	%	Mean	Median	SD
Gender					
Male	27	9.2			
Female	255	87.3			
Other	2	0.7			
Unknown	5	1.7			
Age (in years)			43.24	42	11.64
< 30	55	18.8			
31–40	69	23.6			
41–50	78	26.7			
> 50	89	30.5			
Organization					
Hospital 1	196	67.1			
Hospital 2	95	32.5			
Work experience (in years)			16.85	15	10.70
< 5	49	16.8			
6–12	69	23.6			
13–18	50	17.1			
19–25	61	20.9			
> 25	61	20.9			
Working hours					
Day	97	33.2			
Rotational	193	66.1			
Specialization					
Emergency	24	8.2			
Surgery	28	9.6			
Internal medicine	29	9.9			
Oncology and radiology	37	12.7			
Psychiatry	44	15.1			
Intensive care and anesthesiology	42	14.4			
Pediatrics	25	8.6			
Maternity and gynecology	38	13			
Other	24	8.2			
Profession					
Registered nurse	204	69.9			
Other	38	13			
Licensed practical nurse	27	9.2			
Teaching nurse, assistant nurse manager	23	7.9			
How do you evaluate nursing quality in your unit?			4.03	4	0.79
Poor (Grade 1)	5	1.7			
Satisfactory (Grade 2)	8	2.7			
Good (Grade 3)	33	11.3			
Very good (Grade 4)	174	59.6			
Excellent (Grade 5)	72	24.7			

### 3.2. The Nursing Practice Environment

The nursing staff who responded to the questionnaire gave the nursing practice environment an overall score of 2.58. The highest subscale score was observed for interprofessional collaboration between nurses and physicians (*M* = 3.11, SD = 0.558), while the lowest subscale scores were evident for nurses’ participation in the healthcare organization (*M* = 2.21, SD = 0.558) and adequacy of nurse staffing and resources (*M* = 2.42, SD = 0.64). Other subscales also demonstrated scores that reflected favorable perceptions by the nursing staff, i.e., a score over 2.5; these included foundations of nursing quality (*M* = 2.64, SD = 0.454) along with the leadership abilities, management skills, and support provided by nurse leaders (*M* = 2.54, SD = 0.665) (Table 2).

**TABLE 2:** Mean respondent scores on subscales of the instruments related to nursing practice environment and transformational leadership.

Scale (bold), subscales	Variables	Mean	SD	Min–max	Skewness	Kurtosis	*α*
**Practice Environment Scale-Nursing Work Index (PES-NWI)**	**31**	**2.58**	**0.574**	1.00–4.00			0.788
Nurse participation in hospital affairs (*n* = 292)	9	2.21	0.558	1.00–3.78	0.117	−0.171	0.859
Nursing foundations for quality of care (*n* = 292)	10	2.64	0.454	1.20–3.90	−0.134	0.599	0.790
Nurse manager’s ability, leadership, and support of nurses (*n* = 292)	5	2.54	0.665	1.00–4.00	−0.423	−0.231	0.825
Staffing and resource adequacy (*n* = 292)	4	2.42	0.636	1.00–4.00	−0.013	−0.233	0.814
Collegial nurse–physician relations (*n* = 292)	3	3.11	0.558	1.67–4.00	−0.182	−0.291	0.811
**Transformational Leadership Scale (TLS)**	**54**	**3.36**	**0.969**	1.00–5.00			0.911
Ethical leadership (*n* = 292)	14	3.64	1.043	1.00–5.00	−0.736	−0.237	0.961
Management of the nursing process (*n* = 292)	16	3.37	0.961	1.00–5.00	−0.568	−0.287	0.956
Providing feedback and rewards (*n* = 292)	6	2.91	1.047	1.00–5.00	−0.088	−0.884	0.907
Support for professional development (*n* = 292)	7	3.51	1.022	1.00–5.00	−0.545	−0.409	0.903
Actions of the nurse director (*n* = 288)	11	3.04	0.771	1.00–5.00	−0.067	0.660	0.930

*Note:* PES‐NWI, a four‐point Likert scale (1 = strongly disagree, 4 = strongly agree); TLS, a five‐point Likert scale (1 = strongly disagree, 5 = strongly agree). *α* = Cronbach’s alpha.

Abbreviation: SD = standard deviation.

In exploratory analyses, certain respondent background variables were found to show statistically significant associations with the nursing practice environment. For instance, nurses working regular daytime hours (*M* = 2.33), as well as assistant nurse mangers and teaching nurses (*M* = 2.65), reported a better sense of participation in the healthcare organization. Ratings of interprofessional collaboration between nurses and physicians varied across specializations, with nurses specialized in Oncology and Radiotherapy (*M* = 3.30) and Pediatrics (*M* = 3.28) providing the highest ratings of interprofessional collaboration, while the lowest scores were observed among nurses specialized in Surgery (*M* = 2.88) and Internal Medicine (*M* = 2.87) (Table 3).

**TABLE 3:** Relationships between background variables and assessments of the nursing practice environment subscales (*n* = 292).

Background variable	Nurse participation in hospital affairs	Nursing foundations for quality of care	Nurse manager’s ability, leadership, and support of nurses	Staffing and resource adequacy	Collegial nurse–physician relations
*Gender*					
Male (*n* = 27)	2.13	2.52	2.37	2.47	3.05
Female (*n* = 255)	2.24	2.66	2.57	2.42	3.13
Other (*n* = 2)	—	—	—	—	—
Unknown (*n* = 5)	—	—	—	—	—
	ns	ns	**0.04**	ns	ns

*Age (in years)*					
< 30 (*n* = 55)	2.23	2.65	2.60	2.53	3.15
31–40 (*n* = 69)	2.10	2.62	2.44	2.30	3.07
41–50 (*n* = 78)	2.19	2.63	2.53	2.43	3.14
> 50 (*n* = 89)	2.32	2.66	2.58	2.42	3.09
	ns	ns	ns	ns	ns

*Organization*					
Hospital 1 (*n* = 196)	2.29	2.66	2.67	2.42	3.14
Hospital 2 (*n* = 95)	2.08	2.60	2.26	2.41	3.06
	**0.004**	ns	**<** **0.001**	ns	ns

*Work experience (in years)*					
< 5 (*n* = 49)	2.32	2.67	2.63	2.50	3.09
6–12 (*n* = 69)	2.13	2.63	2.47	2.39	3.15
13–18 (*n* = 50)	2.18	2.68	2.53	2.45	3.12
19–25 (*n* = 61)	2.19	2.56	2.52	2.34	3.04
> 25 (*n* = 61)	2.30	2.66	2.58	2.43	3.13
	ns	ns	ns	ns	ns

*Working hours*					
Day (*n* = 97)	2.33	2.71	2.66	2.47	3.17
Rotational (*n* = 193)	2.16	2.61	2.48	2.39	3.08
	**0.02**	ns	ns	ns	ns

*Specialization*					
Emergency (*n* = 24)	2.13	2.50	2.53	2.20	3.15
Surgery (*n* = 28)	2.14	2.51	2.71	2.23	2.88
Internal medicine (*n* = 29)	2.37	2.71	2.59	2.45	2.87
Oncology and radiology (*n* = 37)	2.34	2.70	2.76	2.50	3.30
Psychiatry (*n* = 44)	2.29	2.62	2.49	2.53	3.04
Intensive care and anesthesiology (*n* = 42)	2.09	2.66	2.30	2.48	3.25
Pediatrics (*n* = 25)	2.43	2.73	2.71	2.39	3.28
Maternity and gynecology (*n* = 38)	2.07	2.58	2.36	2.26	3.10
Other (*n* = 24)	2.14	2.72	2.48	2.59	3.06
	ns	ns	**0.03**	ns	**0.01**

*Profession*					
Registered nurse (*n* = 204)	2.20	2.61	2.52	2.43	3.11
Other (*n* = 38)	2.03	2.62	2.39	2.24	3.15
Licensed practical nurse (*n* = 27)	2.27	2.78	2.47	2.45	3.00
Teaching nurse, assistant nurse manager (*n* = 23)	2.65	2.80	2.98	2.53	3.15
	**<** **0.001**	ns	**0.006**	ns	ns

*How do you evaluate nursing quality in your unit?*					
Poor (Grade 1) (*n* = 5)	1.40	1.58	1.28	1.60	2.73
Satisfactory (Grade 2) (*n* = 8)	1.68	2.14	1.95	1.59	2.38
Good (Grade 3) (*n* = 33)	1.95	2.38	2.18	2.05	2.93
Very good (Grade 4) (*n* = 174)	2.17	2.61	2.50	2.37	3.10
Excellent (Grade 5) (*n* = 72)	2.58	2.96	2.94	2.85	3.32
	**<** **0.001**	**<** **0.001**	**<** **0.001**	**<** **0.001**	**<** **0.001**

*Note:* The data describe answers from a four‐point Likert scale (1 = strongly disagree, 4 = strongly agree). Statistical tests: Independent samples *t*‐test or Mann–Whitney *U* test and one‐way ANOVA or Kruskal–Wallis ANOVA use depended on variable distribution. Statistically significant results are in bold.

Abbreviation: ns = nonsignificant.

Perceptions of nursing leadership abilities, management skills, and support differed between men (*M* = 2.57) and women (*M* = 2.37). Significant differences were also observed across different specializations, with nurses specialized in Oncology and Radiotherapy (*M* = 2.76), Surgery (*M* = 2.71), and Pediatrics (*M* = 2.71) giving the highest ratings of nursing leadership abilities, management skills, and support. Nurses with a specialization of Other (*M* = 2.48), Obstetrics and Gynecology (*M* = 2.36), and Surgery and Intensive Care (*M* = 2.30) gave the lowest ratings of nursing leadership abilities, management skills, and support. A respondent’s profession also affected how they perceived leadership, with teaching nurses and assistant nurse managers giving the highest scores (*M* = 2.98), and radiology nurses, midwives, and public health nurses providing the lowest ratings (*M* = 2.39) of leadership (Table 3).

Statistically significant associations were found between a nurse’s evaluation of unit performance and each subscale related to the nursing practice environment. For instances, nurses who rated their unit’s quality as excellent also provided the highest ratings for subscales related to the nursing practice environment (*M* = 2.58–3.32). In contrast, fair or satisfactory ratings of unit quality returned the lowest scores (*M* = 1.28–2.14) on nursing practice environment subscales. The only exception was interprofessional collaboration between nurses and physicians, which was rated relatively highly regardless of unit performance (*M* = 2.38–3.32) (Table 3).

### 3.3. Transformational Leadership

The nursing staff gave the aspect of transformational leadership an overall score of 3.36. The transformational leadership subscale with the highest score from respondents was ethics in leadership (*M* = 3.64, SD = 1.043), while the subscale of feedback and rewards (*M* = 2.91, SD = 1.047) received the lowest score (Table 2).

In exploratory analyses, statistically significant associations between staff background variables and transformational leadership were observed. For instance, work schedules and professions were linked to the assessment of leadership ethics, namely, daytime workers rated leadership ethics higher (*M* = 3.88) than shift workers (*M* = 3.52). Moreover, teaching nurses and assistant nurse managers rated the transformational leadership of their superiors noticeably higher (*M* = 4.17) than radiology nurses, midwives, and public health nurses (*M* = 3.46). A nurse’s specialization was found to significantly influence how they assess support for professional development, with the highest scores observed among nurses working in Pediatrics (*M* = 3.79) and Psychiatry (*M* = 3.76), while the lowest scores were observed among nurses working in Obstetrics and Gynecology (*M* = 3.17) and Emergency Services (*M* = 3.21) (Table 4).

**TABLE 4:** Relationships between background variables and transformational leadership subscales (n = 292).

Background variable	Ethical leadership	Management of the nursing process	Providing feedback and rewards	Support for professional development	Actions of the nurse director
*Gender*					
Male (*n* = 27)	3.45	3.17	2.71	3.28	2.79
Female (*n* = 255)	3.68	3.40	2.96	3.54	3.07
Other (*n* = 2)	—	—	—	—	—
Unknown (*n* = 5)	—	—	—	—	—
	ns	ns	ns	ns	ns

*Age (in years)*					
< 30 (*n* = 55)	3.77	3.44	2.83	3.53	3.12
31–40 (*n* = 69)	3.52	3.36	2.81	3.39	2.90
41–50 (*n* = 78)	3.64	3.21	2.86	3.41	2.99
> 50 (*n* = 89)	3.66	3.48	3.08	3.69	3.14
	ns	ns	ns	ns	ns

*Organization*					
Hospital 1 (*n* = 196)	3.77	3.48	3.11	3.64	3.14
Hospital 2 (*n* = 95)	3.38	3.16	2.51	3.24	2.86
	**0.02**	**0.01**	**< 0.001**	**0.002**	**0.004**

*Work experience (in years)*					
< 5 (*n* = 49)	3.85	3.45	2.95	3.60	3.05
6–12 (*n* = 69)	3.59	3.38	2.76	3.44	3.06
13–18 (*n* = 50)	3.56	3.34	2.89	3.40	2.99
19–25 (*n* = 61)	3.59	3.23	2.97	3.51	2.91
> 25 (*n* = 61)	3.65	3.47	3.01	3.62	3.18
	ns	ns	ns	ns	ns

*Working hours*					
Day (*n* = 97)	3.88	3.56	3.07	3.68	3.14
Rotational (*n* = 193)	3.52	3.29	2.83	3.43	2.99
	**0.04**	ns	ns	ns	ns

*Specialization*					
Emergency (*n* = 24)	3.66	3.08	2.88	3.21	3.08
Surgery (*n* = 28)	4.09	3.73	3.39	3.73	3.02
Internal medicine (*n* = 29)	3.46	3.42	3.06	3.54	3.12
Oncology and radiology (*n* = 37)	3.83	3.48	3.05	3.67	3.05
Psychiatry (*n* = 44)	3.72	3.39	2.97	3.76	3.08
Intensive care and anesthesiology (*n* = 42)	3.27	3.32	2.61	3.25	2.87
Pediatrics (*n* = 25)	3.93	3.55	3.05	3.79	3.26
Maternity and gynecology (*n* = 38)	3.36	3.08	2.63	3.17	3.06
Other (*n* = 24)	3.61	3.35	2.70	3.54	2.84
	ns	ns	ns	**0.03**	ns

*Profession*					
Registered nurse (*n* = 204)	3.61	3.35	2.83	3.50	3.01
Other (*n* = 38)	3.46	3.10	2.65	3.18	2.90
Licensed practical nurse (*n* = 27)	3.67	3.44	3.16	3.69	3.01
Teaching nurse, assistant nurse manager (*n* = 23)	4.17	3.93	3.83	4.00	3.53
	**0.04**	**0.008**	**< 0.001**	**0.008**	**0.02**

*How do you evaluate nursing quality in your unit?*					
Poor (Grade 1) (*n* = 5)	1.46	1.49	1.50	1.51	1.78
Satisfactory (Grade 2) (*n* = 8)	3.08	2.91	2.46	3.43	2.72
Good (Grade 3) (*n* = 33)	2.97	2.74	2.36	2.90	2.63
Very good (Grade 4) (*n* = 174)	3.62	3.33	2.82	3.46	3.01
Excellent (Grade 5) (*n* = 72)	4.21	3.95	3.56	4.08	3.42
	**< 0.001**	**< 0.001**	**< 0.001**	**< 0.001**	**< 0.001**

*Note:* The data describe answers to a five‐point Likert scale (1 = strongly disagree, 5 = strongly agree). Statistical tests: Independent samples *t*‐test or Mann–Whitney *U* test and one‐way ANOVA or Kruskal–Wallis ANOVA use depended on variable distribution. Statistically significant results are in bold.

Abbreviation: ns = nonsignificant.

Profession was significantly related to ratings of certain transformational leadership subscales. Teaching nurses and assistant nurse managers provided the highest scores for process leadership (*M* = 3.93), feedback and rewards (*M* = 3.83), professional development support (*M* = 4.00), and nurse directors’ performance (*M* = 3.53); at the same time, radiology nurses, midwives, and public health nurses gave the lowest scores for these subscales (Table 4).

The score of each subscale of transformational leadership was significantly associated with a respondent’s evaluation of their unit’s performance. Nurses who rated unit quality as excellent also gave the highest ratings to the subscales of transformational leadership (*M* = 3.26–4.21). In contrast, when the unit quality was poorly rated, the transformational leadership subscales received lower scores (*M* = 1.46–1.78) (Table 4).

### 3.4. Relationship Between Transformational Leadership and Nursing Practice Environment

The presented research found a statistically significant, linear relationship between the overall scores for subscales related to nursing practice environment and transformational leadership. A strong linear association was observed between the subscales of transformational leadership and nurse leaders’ abilities, management skills, and support for nurses in the practice environment (*r* = 0.501–0.786, *p* < 0.001, *r*_*s*_ = 0.507–0.755, *p* < 0.001). A strong linear relationship was also observed between transformational leadership and nurses’ participation in the healthcare organization (*r* = 0.476–0.618, *p* < 0.001, *r*_*s*_ = 0.461–0.605, *p* < 0.001). A moderate linear relationship was found between transformational leadership and interprofessional collaboration between nurses and physicians (*r* = 0.146–0.184, *p* < 0.001, *r*_*s*_ = 0.137–0.204, *p* < 0.001) (Table 5).

**TABLE 5:** Pearson’s correlation coefficients for the relationships between subscales from the practice environment and transformational leadership instruments.

	Ethical leadership	Management of the nursing	Providing feedback and rewards	Support for professional development	Actions of the nurse director
Nurse participation in hospital affairs	0.476^∗∗^	0.601^∗∗^	0.605^∗∗^	0.618^∗∗^	0.573^∗∗^
Nursing foundations for quality of care	0.452^∗∗^	0.605^∗∗^	0.513^∗∗^	0.560^∗∗^	0.454^∗∗^
Nurse manager’s ability, leadership, and support of nurses	0.786^∗∗^	0.762^∗∗^	0.758^∗∗^	0.692^∗∗^	0.501^∗∗^
Staffing and resource adequacy	0.346^∗∗^	0.376^∗∗^	0.360^∗∗^	0.389^∗∗^	0.326^∗∗^
Collegial nurse–physician relations	0.169^∗∗^	0.153^∗∗^	0.147^∗∗^	0.184^∗∗^	0.146^∗∗^

^∗∗^Denotes statistical significance with the threshold set to *p* < 0.001.

Multiple linear regression analysis was used to test the relationships between subscales associated with transformational leadership and the nursing practice environment. The results demonstrated that nurses’ perceptions of transformational leadership show a strong association with their assessments of participation in the healthcare organization, and transformational leadership subscales were related to 54% of the variation in this practice environment subscale. Similarly, transformational leadership subscales were related to 44% of the variation in the foundations of nursing quality (Table 6).

**TABLE 6:** Associations between transformational leadership and perceptions of the nursing practice environment, as determined using linear regression.

Dependent variable	Explanatory variable	*B*	*t*	Cl 95%	*p*
Nurse participation in hospital affairs	(Constant)	0.603	5.647	0.393, 0.813	< 0.001
	Ethical leadership	−0.164	−3.621	−0.253, −0.075	**<** **0.001**
	Management of the nursing process	0.158	2.753	0.045, 0.271	**0.006**
	Providing feedback and rewards	0.116	2.913	0.038, 0.194	**0.004**
	Support for professional development	0.172	3.852	0.084, 0.261	**<** **0.001**
	Actions of the nurse director	0.243	7.312	0.178, 0.308	**<** **0.001**
R Square = **0.540**					

Nursing foundations for quality of care	(Constant)	1.476	15.448	1.288,1.664	< 0.001
	Ethical leadership	−0.144	−3.565	−0.224, −0.065	**<** **0.001**
	Management of the nursing process	0.290	5.647	0.189, 0.391	**<** **0.001**
	Providing feedback and rewards	0.007	0.200	−0.063, 0.077	0.842
	Support for professional development	0.089	2.221	0.010, 0.168	**0.027**
	Actions of the nurse director	0.125	4.199	0.066, 0.184	**<** **0.001**
R Square = **0.442**					

Nurse manager’s ability, leadership, and support of nurses	(Constant)	0.363	3.651	0.167, 0.559	< 0.001
	Ethical leadership	0.299	7.091	0.216, 0.382	**<** **0.001**
	Management of the nursing process	0.077	1.440	−0.028, 0.182	0.151
	Providing feedback and rewards	0.184	4.968	0.111, 0.257	**<** **0.001**
	Support for professional development	−0.045	−1.081	−0.127, 0.037	0.281
	Actions of the nurse director	0.146	4.716	0.085, 0.207	**<** **0.001**
R Square = **0.718**					

Staffing and resource adequacy	(Constant)	1.239	7.686	0.922, 1.557	< 0.001
	Ethical leadership	0.011	0.154	−0.124, 0.145	0.877
	Management of the nursing process	0.045	0.521	−0.125, 0.216	0.603
	Providing feedback and rewards	0.026	0.432	−0.092, 0.144	0.666
	Support for professional development	0.133	1.961	0.000, 0.266	0.051
	Actions of the nurse director	0.148	2.946	0.049, 0.247	**0.003**
R Square = **0.191**					

Collegial nurse–physician relations	(Constant)	2.597	16.917	2.294, 2.899	< 0.001
	Ethical leadership	0.078	1.194	−0.050, 0.206	0.233
	Management of the nursing process	−0.066	−0.801	−0.228, 0.096	0.424
	Providing feedback and rewards	−0.029	−0.515	−0.142, 0.083	0.607
	Support for professional development	0.095	1.473	−0.032, 0.222	0.142
	Actions of the nurse director	0.067	1.406	−0.027, 0.161	0.161
R Square = **0.046**					

*Note:* Statistically significant results are in bold, *B* = unstandardized coefficients, and *p* = *p* value.

Abbreviation: CI, confidence interval.

The analysis revealed that ratings of transformational leadership had the highest explanatory power for assessments of nurse leaders’ abilities, management skills, and support for nurses, with the transformational leadership subscales associated with 72% of the variation. Moreover, positive ratings of process leadership were positively associated with nurses’ perceptions of the foundations of nursing quality (*B* = 0.290, *p* < 0.001), while positive evaluations of nurse directors’ performance showed positive relationships with perceptions of participation in the healthcare organization (*B* = 0.243, *p* < 0.001), the foundations of nursing quality (*B* = 0.125, *p* < 0.001), and the adequacy of nurse staffing and resources (*B* = 0.148, *p* = 0.003) (Table 6). The results of the mixed models were consistent with the linear regression analyses, with similar effect sizes and statistically significant associations (supporting file 1).

## 4. Discussion

The nursing staff who participated in the research provided an overall favorable assessment of the nursing practice environment. Nurses’ perceptions of the quality of the nursing practice environment were comparable to prior studies conducted in Finland and abroad; furthermore, the differences observed between subscales aligned with what has previously been reported [14, 18]. For instance, collaboration between nurses and physicians received the highest ratings in both this study and earlier work. In this study, nurses’ participation in healthcare organization decision‐making received the lowest rating while prior Finnish and international studies have reported nurse staffing and resource adequacy to be lowest‐rated subscale [14, 18].

The nursing staff rated transformational leadership below the Magnet Hospital Model’s excellent leadership threshold of a mean value of 4 out of 5 [19]. In this study, nurse managers’ feedback and reward practices and the performance of nurse directors received the lowest ratings, while leadership ethics and support for professional development were rated the highest. This result mirrors what has been reported in previous studies conducted in Finland and may be associated with cultural and organizational factors inside the specialized healthcare system [19, 21, 22].

The analyses revealed that nurses’ evaluations of transformational leadership and practice environment had statistically significant relationships. Nurses who rated their nurse managers as capable and supportive leaders also provided higher ratings of how their managers lead nursing processes, provide feedback, and practice ethical leadership relative to other responding nurses. The results also indicated that the nurse directors’ leadership practices were related to nurses’ perceived opportunities to participate in organizational decision‐making. It has been identified that nurse leaders play a critical role in engaging clinical nurses in the development of healthcare organizations by providing strong support and the necessary resources while dismantling hierarchical barriers in decision‐making processes [49].

Although previous studies have examined the nursing practice environment and transformational leadership separately and reported associations with nursing‐related outcomes, our literature review suggests that limited research has addressed the relationship between these constructs in specialized healthcare settings [6, 18]. The present study contributes to this body of knowledge by demonstrating an association between nurse leaders’ transformational leadership practices and perceptions of the quality of the nursing practice environment. Labrague et al. [27] previously found a relationship between authentic leadership and the nursing practice environment, while Numminen et al. [14] also identified the significance of nurse leaders in achieving optimal practice environments. Nurse leaders should establish clear goals, treat employees equitably in terms of workload and professional development opportunities, and consistently strive for excellence within their units in specialized healthcare. These factors are key to fostering a culture in which staff members will adopt these values and perceive that the practice environment is functioning well [14, 27].

In this study, nurses who provided favorable assessments of the nursing practice environment also rated the quality of care in their specific unit as excellent. Assessments of nursing care quality improved when appropriate facilities and equipment were available and when there were sufficiently skilled colleagues present at every shift. Nurse directors’ actions were significantly associated with the evaluation of nurse staffing and resource adequacy, highlighting the important role of nurse leaders in these aspects of nursing work. Previous studies have also established that nurse staffing and resource adequacy are linked with the quality of nursing care based on assessments by both nurses and patients [11–13, 27, 32–34, 50]. As such, it comes as no surprise that nursing researchers and practitioners argue that nurses should be regarded as a valuable human capital investment rather than merely a cost item on the financial statement [3].

Earlier research has also linked transformational leadership to nursing care quality. This is because clear transformational leadership among nurse managers is associated with factors such as medication safety, the occurrence of adverse events, and patient evaluations of care quality [19, 22, 26, 42]. Research by Lappalainen et al. [22] found that certain subdimensions of transformational leadership, more specifically, process management by nurse managers, show positive associations with nurses’ perceptions of medication safety in their unit. The results also demonstrated that scores on subdimensions of transformational leadership were related to nurses’ evaluations of nursing care quality at their unit [22]. In this study, more favorable assessments of transformational leadership were also associated with higher ratings of care quality at the unit level. The results, both those shared in this paper and what has been previously reported, emphasize how nurse managers play a critical role in fostering a culture of safety and enabling structures for high‐quality patient care [22, 32, 42].

Nurses in the present study generally rated the nursing practice environment favorably, although participation in healthcare organizations and the adequacy of nurse staffing and resources were evaluated less positively, consistent with previous international findings identifying these as the weakest subdimensions [18, 50, 51]. Given that a supportive practice environment is linked to nurse recruitment and retention, these areas should be prioritized to address workforce shortages [27, 32–34]. Similarly, all subdimensions of transformational leadership scored below the Magnet model’s threshold for excellence (≥ 4), with particularly low ratings for feedback, reward practices, and nurse directors’ performance, in line with earlier Finnish studies [19, 21, 22]. These findings highlight the need for nurse leaders to strengthen individualized support and professional development opportunities, which may be constrained by wide spans of control and the organizational demands placed on nurse leaders [52, 53].

The results presented in this study indicated that the transformational leadership practices of nurse leaders were related to other nurses’ perceptions of the quality of the nursing practice environment. The results also showed that the participating nurses valued the actions of nurse directors as well as their support. As such, nurse directors and nurse managers must be present, treat employees equitably and fairly, maintain regular communication, and advocate for clinical nurses within healthcare organizations. Nurses should also be empowered to make decisions about their own work. As described previously by Nurmeksela et al. [53], each healthcare organization must include the necessary structures to enable nurse leaders to demonstrate excellent leadership.

### 4.1. The Applicability of the Results in Nursing Practice

The results of this study can be utilized by healthcare organizations to develop nursing practice environments and transformational leadership among nurse leaders. A well‐rated nursing practice environment, along with transformational leadership actions by nurse leaders, will enhance nurses’ job satisfaction and well‐being, improve the quality of patient care, and strengthen organizational attractiveness and retention. All of these are crucial to the sustainable management of a healthcare organization. The findings of this study provide a blueprint for identifying the aspects of leadership and working conditions that healthcare organizations and nurse leaders should focus on improving.

### 4.2. Study Limitations

The presented research was characterized by a low response rate (7%), and the number of respondents (*n* = 292) fell below the threshold required for generalizing the results to the target population. Furthermore, because no data on nonrespondents were available, the possibility of substantial nonresponse bias cannot be excluded, and conclusions should be drawn from the results with caution. Low response rate aligns with the European cross‐sectional survey trend among nursing profession [54] and should consider the fact that the data were collected at the end of the COVID‐19 pandemic and in the middle of Finnish national healthcare reform that might explain the low response rate. Target population of this study was also small and clearly defined. The study employed a discretionary sampling approach to specialized Finnish healthcare units that are applying for Magnet hospital status; this means that the results cannot be generalized to all Finnish nurses or other types of healthcare settings [46].

Although mixed models were tested to account for clustering at the hospital level, the number of clusters was small (two hospitals), which limits the reliability of random effect estimation. Therefore, the mixed model results should be interpreted as supportive rather than definitive. Furthermore, although regression analyses were conducted to examine associations between study variables, multicollinearity among transformational leadership subscales (VIF 1.293–5.994) may have influenced individual regression coefficients, including the occurrence of negative coefficients despite positive bivariate correlations. Therefore, individual coefficients should be interpreted with caution [55]. Finally, the regression models were not adjusted for respondent characteristics, such as profession, specialty, or work schedule. Given the observed differences in the study outcomes across these characteristics, residual confounding cannot be ruled out.

## 5. Conclusion

Providing feedback and rewards was perceived as the weakest aspect of transformational leadership among Finnish Magnet‐aspiring specialized healthcare units. This finding identifies feedback and recognition practices, including appropriate rewards, competence‐based pay structures, and regular, constructive feedback on work performance, as potential areas for further development. Leadership actions at higher organizational levels in specialized healthcare were perceived as distant by clinical nurses, suggesting a potential need for increased leader visibility and more direct interaction with frontline staff. The findings also highlight adequate resources, functional systems, appropriate facilities, and sufficient clinically competent staffing as areas requiring attention. Furthermore, supporting nurses’ participation in work‐related decision‐making and fostering continuous professional development may be relevant considerations in efforts to maintain high‐quality nursing practice.

### 5.1. Recommendations for Further Research

The conclusions and limitations of the present study suggest that the nursing practice environment should be investigated further in Finland—via broader data collection from various nursing units—as this is currently a relatively under‐researched area. Additionally, the impact of different leadership styles on how clinical nurses evaluate the nursing practice environment should be explored through longitudinal studies and intervention research designs. The leadership activities of nurse directors and chief nursing officers should also be studied to better understand the specific effects of their leadership practices.

### 5.2. Implications for Nursing Management

The findings of this study can guide Magnet‐aspiring specialized healthcare organizations in developing the nursing practice environment and the transformational leadership practices of nurse leaders. The present study found an association between positive perceptions of transformational leadership among nurse leaders and positive perceptions of the nursing practice environment. Previous research has linked these aspects with important outcomes in nursing including nurses’ job satisfaction and well‐being, quality of care, and organizational attraction and retention. Although these outcomes were not examined in the present study, the findings may help healthcare organizations and nurse leaders identify aspects of leadership and working conditions that warrant attention when developing the nursing practice environment and leadership practices.

## Funding

There was no source of funding.

## Conflicts of Interest

The authors declare no conflicts of interest.

## Supporting Information

Additional supporting information can be found online in the Supporting Information section.

## Supporting information


**Supporting Information 1** Supporting file 1 presents the results of linear mixed models conducted as a sensitivity analysis to account for potential clustering at the hospital level.

**Supporting Information 2** STROBE checklist.

## Data Availability

Data are available on request from the authors.
