# Supplementary material for: Relationships Between Transformational Leadership and the Nursing Practice Environment: A Cross‐Sectional Study
Source: J Nurs Manag. 2026 Nov 8;2026:9298780. doi: 10.1155/jonm/9298780 (PMC13745989; doi:10.1155/jonm/9298780)
Supplement: Supplementary file 1 [file JONM-2026-9298780-s001.docx]

Supplementary file 1: The effects of transformational leadership on perceptions of the nursing practice environment, as determined using linear mixed models

| **Dependent variable** | **Variable** | **B** | **SE** | **Cl 95 %** | **p value** |
| --- | --- | --- | --- | --- | --- |
| Nurse participation in hospital affairs |  |  |  |  |  |
|  | (Intercept) | 0.604 | 0.107 | 0.393, 0.814 | <0.001 |
|  | Ethical leadership | -0.164 | 0.045 | -0.253, -0.075 | **<0.001** |
|  | Management of the nursing process | 0.159 | 0.057 | 0.046, 0.272 | **0.006** |
|  | Providing feedback and rewards | 0.116 | 0.040 | 0.037, 0.194 | **0.004** |
|  | Support for professional development | 0.172 | 0.045 | 0.084, 0.261 | **<0.001** |
|  | Actions of the Nurse Director | 0.243 | 0.033 | 0.178, 0.308 | **<0.001** |
| Nursing Foundations for Quality of Care |  |  |  |  |  |
|  | (Intercept) | 1.479 | 0.096 | 1.289, 1.668 | <0.001 |
|  | Ethical leadership | -0.144 | 0.040 | -0.224, -0.065 | **<0.001** |
|  | Management of the nursing process | 0.292 | 0.051 | 0.191, 0.393 | **<0.001** |
|  | Providing feedback and rewards | 0.006 | 0.036 | -0.064, 0.077 | 0.857 |
|  | Support for professional development | 0.090 | 0.040 | 0.011, 0.169 | **0.026** |
|  | Actions of the Nurse Director | 0.122 | 0.030 | 0.063, 0.181 | **<0.001** |
| Nurse manager’s ability, leadership, and support of nurses |  |  |  |  |  |
|  | (Intercept) | 0.391 | 0.119 | 0.115, 0.668 | 0.012 |
|  | Ethical leadership | 0.294 | 0.042 | 0.212, 0.376 | **<0.001** |
|  | Management of the nursing process | 0.094 | 0.053 | -0.010, 0.199 | 0.076 |
|  | Providing feedback and rewards | 0.163 | 0.037 | 0.090, 0.236 | **<0.001** |
|  | Support for professional development | -0.045 | 0.041 | -0.126, 0.036 | 0.278 |
|  | Actions of the Nurse Director | 0.136 | 0.031 | 0.076, 0.197 | **<0.001** |
| Staffing and resource adequacy |  |  |  |  |  |
|  | (Intercept) | 1.230 | 0.167 | 0.895, 1.565 | <0.001 |
|  | Ethical leadership | 0.013 | 0.068 | -0.121, 0.147 | 0.850 |
|  | Management of the nursing process | 0.041 | 0.087 | -0.130, 0.212 | 0.639 |
|  | Providing feedback and rewards | 0.035 | 0.061 | -0.085, 0.155 | 0.565 |
|  | Support for professional development | 0.134 | 0.068 | 0.001, 0.267 | **0.049** |
|  | Actions of the Nurse Director | 0.147 | 0.051 | 0.048, 0.247 | **0.004** |
| Collegial nurse-physician relations |  |  |  |  |  |
|  | (Intercept) | 2.598 | 0.154 | 2.295, 2.900 | <0.001 |
|  | Ethical leadership | 0.078 | 0.065 | -0.051, 0.206 | 0.234 |
|  | Management of the nursing process | -0.065 | 0.083 | -0.228, 0.098 | 0.431 |
|  | Providing feedback and rewards | -0.030 | 0.057 | -0.143, 0.083 | 0.603 |
|  | Support for professional development | 0.095 | 0.065 | -0.032, 0.222 | 0.142 |
|  | Actions of the Nurse Director | 0.066 | 0.048 | -0.029, 0.161 | 0.171 |

Statistically significant results are in bolded. B = Estimate, SE = Standard error, CI = confidence interval, p = p-value

Note: Hospital I & II as Random variance, TLS subscales as Fixed effects, the between-hospital variance was negligible across all models (variance ≈ 0), indicating minimal clustering effects.
